# Long-term follow-up of the medial arch correction with calcaneal medialization osteotomy in progressive collapsing foot deformity

**DOI:** 10.1007/s00264-025-06464-w

**Published:** 2025-03-04

**Authors:** Julien Farge, Aurélien Moulin-Traffort, Romain Derousseaux, Valentin Rodrigues, Carlos Maynou, Thomas Amouyel

**Affiliations:** 1 CHU Lille, Service d’Orthopédie 1, Lille, France; 2 University of Lille, Lille, France; 3 Univ. Lille, CNRS, INSERM, CHU Lille, Institut Pasteur de Lille, UMR9020-U1277 - CANTHER - Cancer Heterogeneity Plasticity and Resistance to Therapies, Lille, France

**Keywords:** Flatfoot, Calcaneal osteotomy, Adult-acquired flatfoot deformity, Hindfoot valgus

## Abstract

**Purpose:**

Medializing displacement calcaneal osteotomies are part of conservative surgical treatments and represent a reliable option in valgus flatfoot deformities. Favorable short-term results of this procedure is well-known. However, there are few series with follow-up beyond five years. This study reports the clinical outcomes of calcaneal medialization osteotomy with a minimum follow-up of five years. The primary objective was to compare functional scores and radiographic measurements at the preoperative stage, immediately post operative, and at the final follow-up.

**Materials and methods:**

This was a retrospective, single-centre, multi-operator study of 32 patients, who underwent a medialization calcaneal osteotomy for type II flatfoot. Clinical evaluation of the patients was conducted using the American Orthopaedic Foot and Ankle Surgery (AOFAS) score and the European Foot and Ankle Society (EFAS) score. Radiographic evaluation used the plantar arch angle angle, the talus–first metatarsal axis (T-M1) on lateral weight-bearing radiographs, and calcaneal valgus on Meary’s angle in a hindfoot alignment view.

**Results:**

Mean follow-up was seven years. AOFAS score improved from 46 to 87 and EFAS score from 11 to 20 (*p* < 0.05). Each radiographic parameter was significantly modified between the preoperative and immediate postoperative periods.

**Conclusion:**

We observed a significant and lasting improvement in functional scores at a mean follow-up of seven years. The correction of the evaluated radiographic parameters was significant and remained stable over time.

## Introduction

Flatfoot deformity is a common foot condition in the general population. Estimated prevalence in adults ranges between 15% and 25% [1, 2]. Medializing calcaneal osteotomies are part of conservative surgical treatments and represent a reliable option [3]. In the management of flexible flatfoot, Myerson et al. combined a transfer of the flexor digitorum longus with calcaneal osteotomy [4, 5]. The favourable outcomes of this intervention, both in terms of pain relief and improvement in foot alignment, are well-documented in the short and medium term [3–6]. However, there are few series with follow-up extending beyond five years. Studies typically focus on osteotomy combined with a transfer [4–6], with limited data on isolated osteotomies [7]. Long-term data on surgical outcomes are limited, particularly regarding subtalar joint changes and the frequency of revision surgeries with additional arthrodesis procedures [8]. This study reports the clinical outcomes of calcaneal medialization osteotomy with a minimum follow-up of five years. The primary objective was to compare functional scores and radiographic measurements at the pre-operative stage, immediately post-operative, and at the final follow-up. Secondary objectives focused on assessing complications, long-term arthritic deterioration in the operated foot, and the requirement for post-operative orthopaedic insoles.

## Materials and methods

We conducted a retrospective, single-centre, multi-operator study. We included all patients who underwent a medialization calcaneal osteotomy for type II flatfoot according to Bluman classification [9]. Additional surgical procedures could be performed during the same operative time, including Achilles tendon lengthening, flexor digitorum longus tendon transfer or flexor hallucis longus tendon transfer, excision of an accessory navicular bone, and gastrocnemius aponeurotomy. Minimum follow up time was five years.

Fifty-five patients, accounting for 62 feet, were included in the study. Thirty-two patients attended the follow-up consultation, including six who had bilateral procedures; 16 were lost to follow-up, of whom one had died; and seven declined to participate in the study. All patients were operated between January 2011 and December 2018 by senior surgeons from the department.

Data were collected from imaging records and during dedicated consultations for this study from November 2022 and May 2023 by an independent observer. Clinical evaluation of the patients was conducted using the American Orthopaedic Foot and Ankle Surgery (AOFAS) score and the European Foot and Ankle Society (EFAS) score [10].

**Fig. 1:**
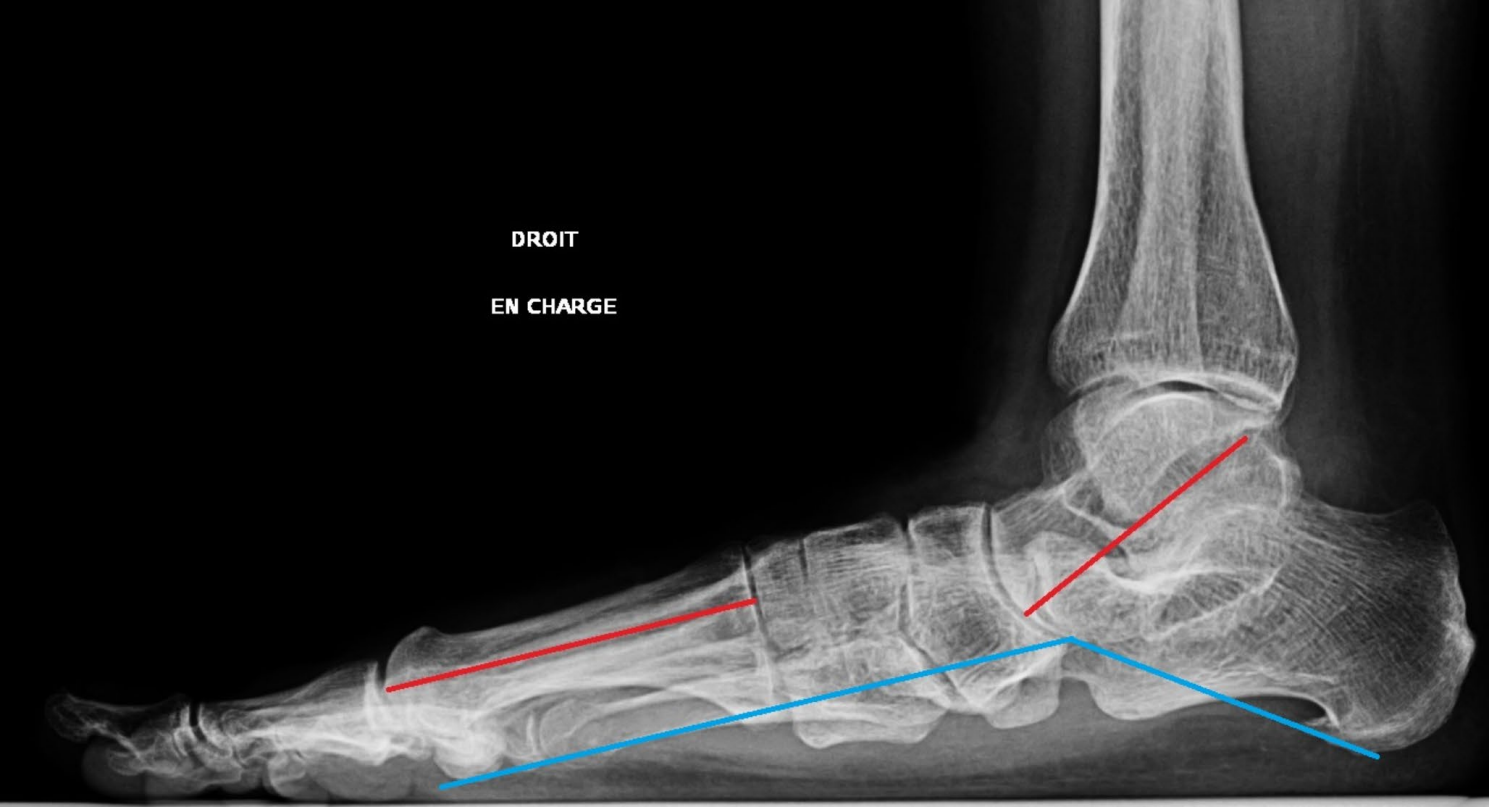
Lateral weight-bearing radiograph of the right foot. Talus–first metatarsal axis in red and plantar arch angle in blue

Three commonly used parameters for assessing flatfoot were measured pre-operatively, post-operatively, and at the last follow-up [11]: the plantar arch angle (Djian-Annonier angle) (between the lowest point of the calcaneus, the lowest point of the talonavicular joint, and the lowest point of the medial sesamoid), the talus–first metatarsal axis (T-M1) on lateral weight-bearing radiographs (Fig. 1), and calcaneal valgus on Meary’s angle in a hindfoot alignment view.

### Surgical procedure

The patient was positioned in lateral decubitus, stabilized with a dorsolumbar support and a pubic support. A tourniquet was applied and inflated either at the upper part of the thigh or at the ankle, depending on the type of anaesthesia (general or regional).

**Fig. 2:**
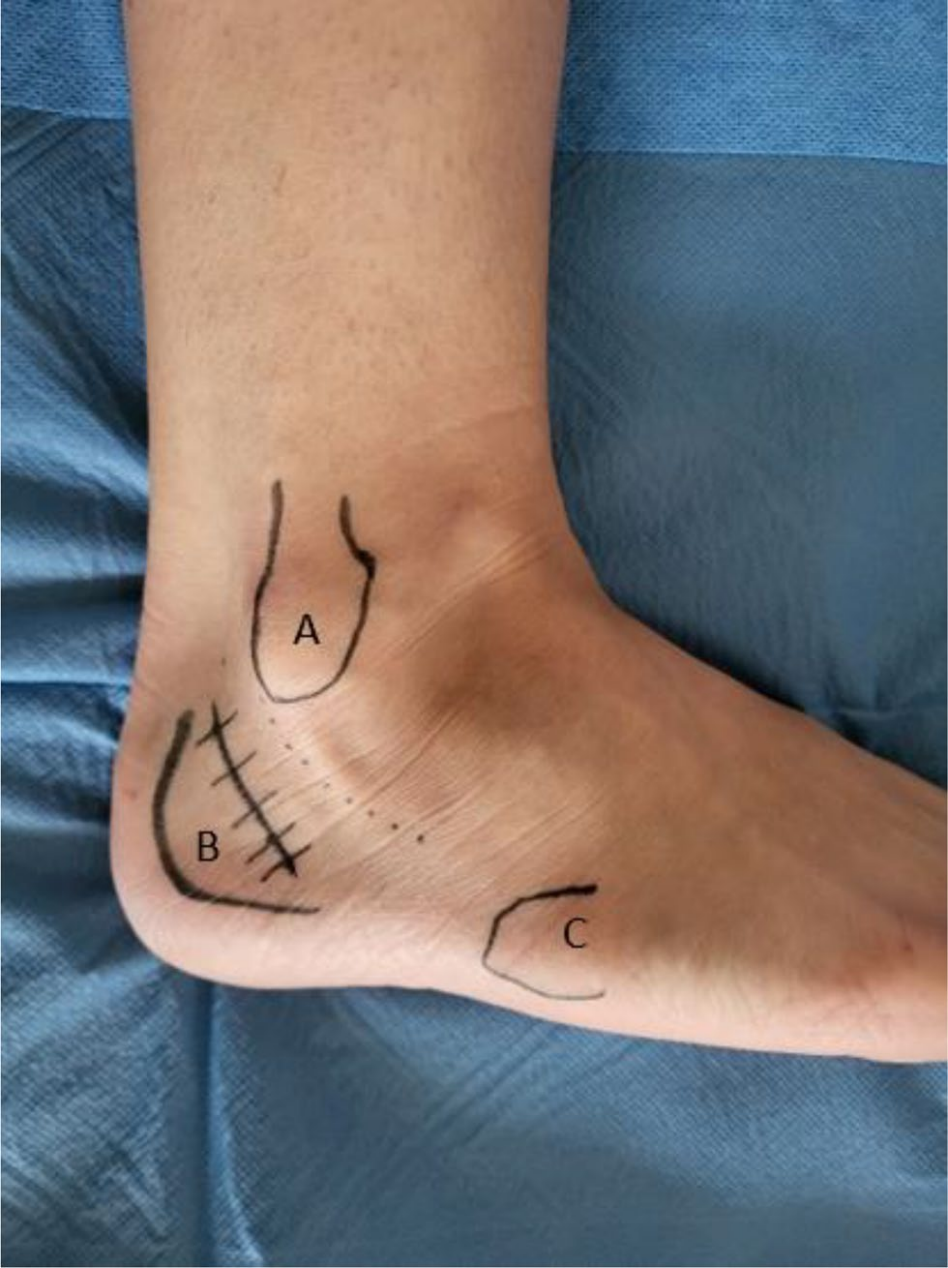
Lateral approach and anatomical landmarks. **A** Lateral malleolus. **B** Posterior edge of the calcaneum. **C** Base of fifth metatarsal

The skin incision was oblique from back to front, posterior to the lateral malleolus, to respect the neurological “safe zone” described by Park et al. [12]. Subcutaneous dissection to access the lateral aspect of the calcaneus was limited to preserve skin vascularization (Fig. 2).

The osteotomy was performed using an oscillating saw after fluoroscopic control, with the cut being oblique and perpendicular to the axis of the calcaneal tuberosity.

**Fig. 3:**
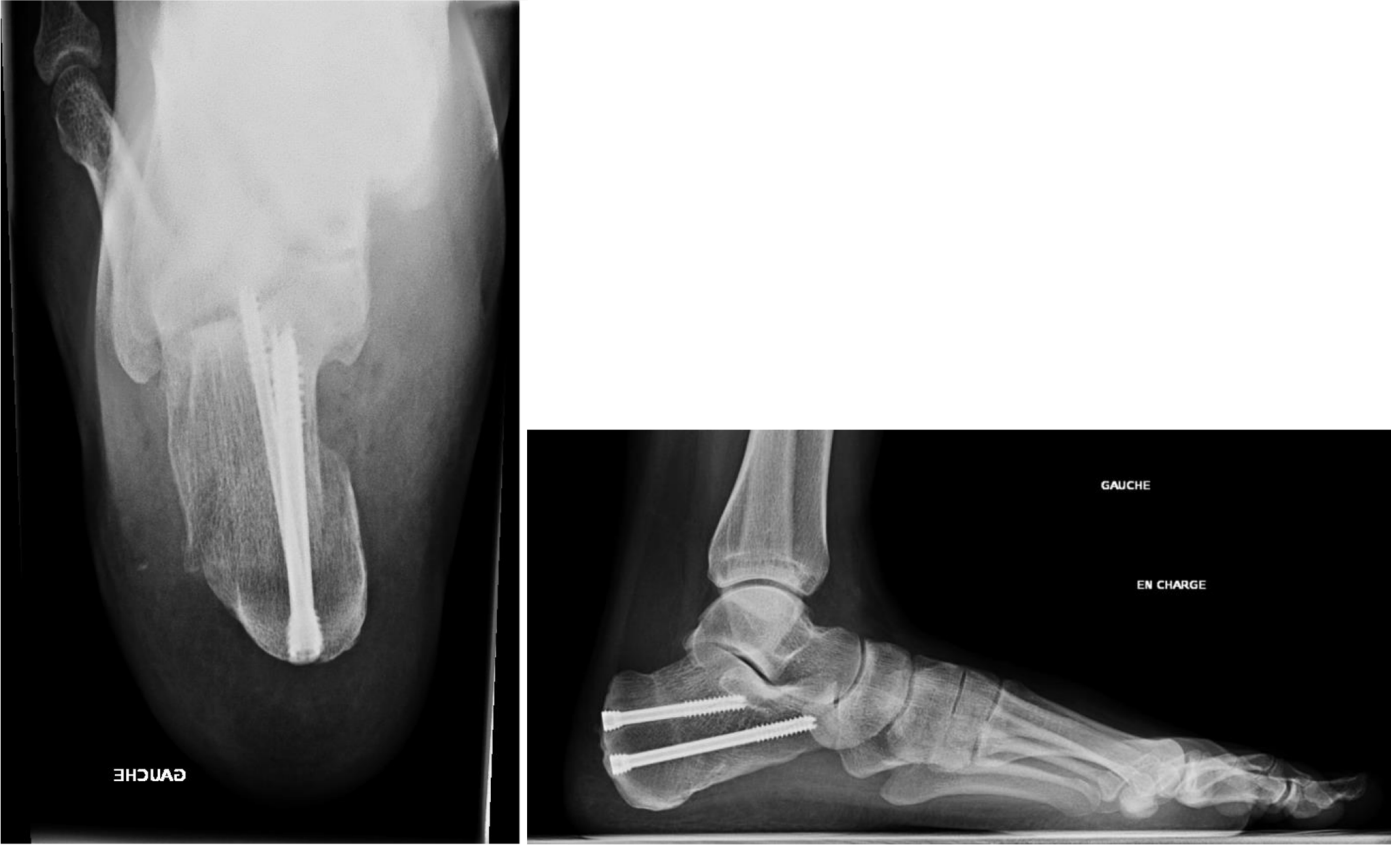
Calcaneal osteotomy stabilized by two talar screws; lateral weight bearing view and retrotibial view

**Fig. 4:**
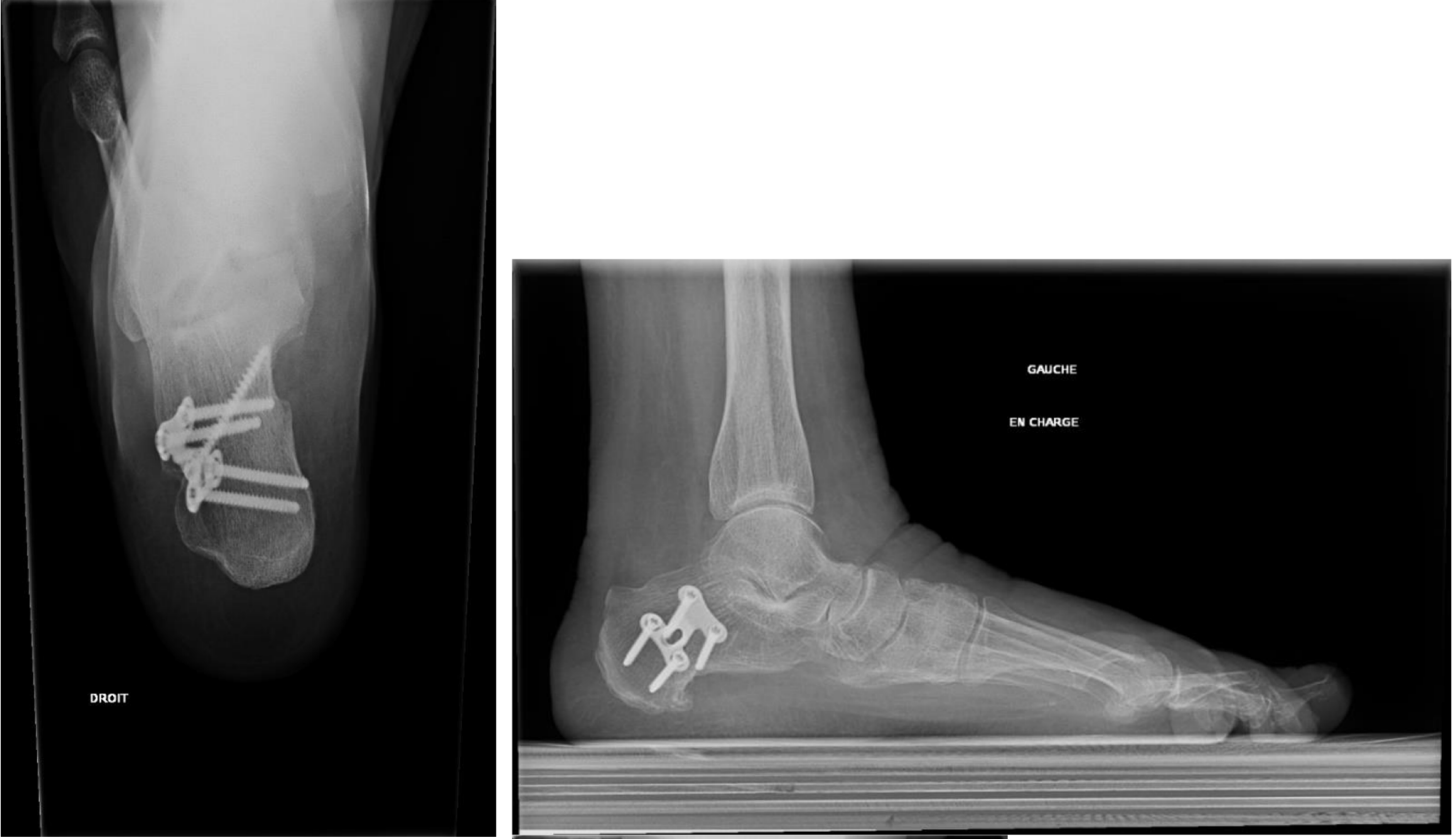
Medial displacement osteotomy stabilized by lateral plate; weight bearing lateral view and retro tibial view

Osteosynthesis was carried out with Large Qwix^®^ self-compressing screws (Newdeal, Saint-Priest, France) in nine patients. Dedicated Step Plate^®^ plates (Arthrex, Naples, FL, USA) were used in 29 procedures (Figs. 3 and 4).

When a tendon transfer was performed, the patient was repositioned in a supine position. The medial approach was oblique, centered on the posterior tibial tendon (PTT) and its insertion on the navicular bone. After opening the tendon sheath and exploring the tendon, the flexor digitorum longus was cut at the level of Henry’s knot and freed distally. A dorsoplantar tunnel was then created in the navicular bone. The graft was passed from plantar to dorsal.

If the graft length was adequate, the tendon was secured to itself. In cases of a short graft, Mitek G4^®^ transosseous anchors (Depuy Mitek, Norwood, MA) were used. In all cases, the foot was maintained in forced inversion during fixation to ensure maximum tension of the tendon transfer.

Data were analyzed using R statistical software version 4.3.1. Patient characteristics were described according to their nature, either by mean and standard deviation or by counts and percentages.

Variables of interest were compared between pre-operative and post-operative periods using the paired Student’s t-test for parametric data or the paired Wilcoxon test for non-parametric data.

Unpaired tests were performed to compare variables according to other factors (e.g., patients with and without tendon transfer, calcaneal valgus greater or less than 15°, etc.). For categorical variables, we performed the parametric chi-squared test, or Fisher’s exact test if conditions were not met. For continuous variables, the Student’s t-test was used in case of normal distribution; otherwise, the non-parametric Wilcoxon test was applied. When both variables were continuous, Kendall’s correlation test was used.

The threshold for statistical significance was set at *p* < 0.05.

## Results

**Table 1:** Descriptive and clinical characteristics of the population

	*n* = 32
Age,mean (SD)	52,8 (9.7)
Sex, n (%)	
Male	5 (16%)
Female	27 (84%)
Side, n (%)	
Right	22 (58%)
Left	16 (42%)
Smoker, n (%)	1 (3%)
Diabetic, n (%)	3 (10%)
BMI, mean (SD)	27,7 (6,2)

The characteristics of the population are reported in Table 1.

Seven patients had previously undergone foot or ankle surgery: hallux valgus correction in four cases; one for the management of a bimalleolar fracture; and two for transverse tarsal joint arthrodesis during childhood.

The mean follow-up was seven years (range 5.0–12.1). Regarding operative data parameters, mean translation was 8.6 mm (SD = 1.9). 18 patients underwent flexor digitorum longus transfer, one had hallux longus flexor transfer, 16 had Achilles tendon lengthening, one underwent gastrocnemius aponeurotomy, three had posterior tibialis synovectomy and one had accessory navicular bone resection.

In cases of isolated osteotomy without tendon transfer or Achilles tendon lengthening, the average operative time was 45 min (SD = 15). If a tendon transfer was performed, the procedure lasted an average of 78 min (SD = 13).

Fixation was achieved with screws in nine cases and with a lateral plate in 29 cases. No statistically significant difference in postoperative outcomes was found between these two groups.

**Table 2:** Preoperative and final Follow-up clinical scores

	Preoperative *n* = 38	Last follow-up *n* = 38	*p*.value	test
AOFAS Pain	7.63 (SD = 10.25)	33.16 (SD = 9.33)	< 0.001	Paired Wilcoxon
AOFAS Function	35.71 (SD = 7.31)	46.29 (SD = 4.42)	< 0.001	Paired t-test
AOFAS Alignment	3.82 (SD = 2.15)	5.26 (SD = 2)	0.001089	Paired Wilcoxon
AOFAS Total	46.68 (SD = 15.94)	83.66 (SD = 13.51)	< 0.001	Paired t-test
EFAS	11.2 (SD = 3.83)	20.08 (SD = 3.24)	< 0.001	Paired t-test

The pre- and post-operative AOFAS and EFAS clinical scores are provided in Table 2. There is a statistically significant improvement for all parameters.

Each radiographic parameter was significantly modified between the pre-operative and immediate post-operative periods.

At a follow-up of more than five years, no significant differences were found between the immediate post-operative measurements and those taken at the final follow-up.

All results regarding the evolution of radiographic parameters are summarized in Table 3.

**Table 3:** Evolution of radiographic parameters between preoperative and immediate postoperative, and between immediate postoperative and Follow-up consultation. Evaluation according to the Wilcoxon test

	Preoperative *n* = 38	Postoperative *n* = 38	*p*.value
Plantar arch angle	132.61 (SD = 5.64)	128.28 (SD = 5.18)	<0.001
Calcaneal valgus	12.11 (SD = 2.3)	8.29 (SD = 2.79)	<0.001
Talus-M1	10.08 (SD = 6.95)	7.70 (SD = 4.77)	0.000052
	**Postoperative** ***n*** ** = 38**	**Last follow-up** ***n*** ** = 38**	***p*** **.value**
Plantar arch angle	128.28 (SD = 5.18)	128.5 (SD = 6.13)	0.546331
Calcaneal valgus	8.29 (SD = 2.79)	8.74 (SD = 2.99)	0.163568
Talus-M1	7.70 (SD = 4.77)	8.6 (SD = 6.23)	0.516814

The analysis of correlations between pre-operative radiographic parameters and post-operative clinical scores did not reveal any significant relationships. Kendall’s Tau values were − 0.01 and 0.02 (*p* > 0.05) for the AOFAS score concerning preoperative calcaneus valgus and Talus–M1 measurements, respectively. Comparable values were found for the EFAS score.

Similarly, no statistical correlation was found between post-operative radiographic criteria and post-operative scores.

Seven patients (18%) reported persistent discomfort in the form of painful paraesthesia or anaesthesia on the lateral side of the foot in the sural nerve territory. Among them, four patients had a scar outside the “safe zone.” Conversely, seven patients had a scar outside the “safe zone” without associated pain.

We had no cases of deep infection or osteotomy non-union. Only two patients, operated on with lateral plates, experienced delayed healing, resolved with local care for one and plate removal at six months for the other. Six patients, three treated with plates and threewith screws, (15%) required removal of their hardware at an average of 12.8 months.

Among the 19 patients who underwent tendon transfer, 13 of them (68%) still required insoles post-operatively. In the group without tendon transfer, 11 patients (57%) still needed insoles after the surgery. This difference is not statistically significant (*p* = 0.7366).

## Discussion

This sample is comparable to other populations described in the literature like Osbeck et al. [13].

The clinical scores in our series showed a significant improvement after the surgery, with an AOFAS score of 83.66 and an EFAS score of 20.08. Myerson et al. reported similar values, with an AOFAS score of 79 points [5].

There was no significant improvement in clinical scores between groups when the calcaneal translation was greater than or equal to 10 mm as compared to a 7.5 mm translation achieved with Step Plate^®^ plates.

In this study, six patients were unsatisfied with the surgery, with AOFAS scores below 70 points and EFAS scores below 16, representing 14% of our series. Persistence of pain and associated functional limitations were the main reason for patients’ dissatisfaction with the surgical outcome. Myerson et al. in 2002 reported an 8% dissatisfaction rate post-operatively for similar reasons [5]. Fayazi et al. reported that two patients (9%) did not improve: one due to a work-related injury and the other due to deep vein thrombosis with residual leg oedema.

Among our six dissatisfied patients, one experienced a recurrence of the flatfoot valgus deformity, while another showed no correction of a significant deformity, with a calcaneal valgus of 14° and a talus–M1 angle of 16° pre-operatively. A third patient is being treated for chronic pain in the operated limb. For the remaining patients, no obvious cause for the persistent pain was identified, which does not appear to be related to iatrogenic sural nerve damage or ankle osteoarthritic deterioration.

At the last follow-up, radiographic parameters remained unchanged from those recorded immediately post-operatively. These results are consistent with the study by Niki et al. in 2012 [3], which highlighted the persistence of radiographic changes between the immediate post-operative period and the final follow-up. However, they noted a loss of correction in patients with a calcaneal valgus > 15°, who also had poorer functional scores. In our study, only six patients had such a valgus, and no significant differences were found between the two groups. However, we observed that these patients had the least satisfactory scores, with under-correction or recurrence of the deformity, even though the intraoperative translation performed was 7.5–10 mm.

A pre-operative lateral talus–M1 angle greater than 25° is associated with poorer long-term outcome [3]. None of our patients presented with such a deformity, which probably supports a better pre-operative selection, excluding Myerson medialization osteotomy in the most severe cases.

We did not find any correlation between pre- or post-operative radiographic measurements and the clinical outcomes reported by the patients. This observation aligns with literature data from Niki and Sammarco on Myerson osteotomy, which does not significantly modify the structural parameters of the foot [3, 14].

When analyzing patients by the tendon transfer status, the demographic groups were similar apart from age. Patients who underwent a transfer were older than those who did not (49.3 vs. 56.3, *p* = 0.024).Post-operatively, the clinical outcomes were comparable between the groups with and without tendon transfer. DiDomenico et al. previously reported that satisfactory results could be achieved without tendon transfer, but the surgical technique described combined a double calcaneal osteotomy, first column stabilization, and gastrocnemius lengthening [15].

Radiographically, the minimal impact of tendon transfer on arch correction is well-known, with structural changes mainly attributed to the osteotomy [4, 16].

In 2007, Bolt compared calcaneal medialization and lengthening osteotomies, demonstrating a greater and more lasting correction with lengthening, but at the cost of increased adjacent joint osteoarthritis and more frequent nonunion. To the best of our knowledge, no studies have employed a follow-up period as lengthy as that observed in isolated medialization osteotomies with or without tendon transfer.

The combination of lengthening and medialization osteotomies has been suggested by some authors to correct both heel valgus and forefoot abduction, especially when talar uncovering exceeds 30° [17, 18]. The available two year results are favorable, with an AOFAS score around 80 and a restoration of medial arch height on radiographs [18].

Osman et al. reported that hardware discomfort was the main reason for reoperation, which was performed in 26% of their patients [19, 20]. In our series, the reoperation rate was lower, with only 15% requiring hardware removal, with no difference between plates and screws.

Six patients experienced pain during clinical examination of the subtalar joint, raising concerns about potential osteoarthritic degeneration. However, this degeneration appears to be relatively well-tolerated, as none of the patients had sought specific treatment for these pains, such as corticosteroid injections.

In the two published series with follow-ups of more than ten years concerning medialization osteotomy, the reoperation rate was low. In Ruffilli’s series of 90 medialization osteotomies, four failures (4.5%) required revision surgery with subtalar arthrodesis [21]. Chadwick et al. also noted four failures (12%), defined as a recurrence of pain and loss of function requiring revision surgery.

To date, none of our patients has required revision surgery for hindfoot arthrodesis, which is the alternative to conservative treatment with calcaneal osteotomies [22, 23].

Ivanic *and al.* and Gonzalez-Martin *and al.* report a neurological complication rate between 7% and 35% [24, 25].

The development of percutaneous surgery could lower neurological complications, but there is a discrepancy on this point in the literature [26–28]. Wayzi et al. in 2018 showed a shorter operating time, but similar X-ray irradiation and no significant difference in the clinical outcome.

In this study almost 40% of the initial operated patients were lost to follow up over five years, but given this long term this figure can be considered as acceptable [29].

Regarding the analysis of radiographic parameters, we must note the absence of an evaluation of the talo-navicular divergence on the weight-bearing anteroposterior foot radiograph. This parameter is frequently assessed as midfoot abduction; however, the lack of systematic preoperative imaging made it impossible to compare findings before and after surgery.

## Conclusion

The Myerson medialization osteotomy for the management of patients with flexible flatfoot and associated posterior tibial dysfunction is a procedure with reliable long-term results. While functional scores are satisfactory, the structural correction of the medial remains unchanged and durable over time.

## Data Availability

No datasets were generated or analysed during the current study.
